# Case Based Measles Surveillance in Pune: Evidence to Guide Current and Future Measles Control and Elimination Efforts in India

**DOI:** 10.1371/journal.pone.0108786

**Published:** 2014-10-07

**Authors:** Anindya Sekhar Bose, Hamid Jafari, Stephen Sosler, Arvinder Pal Singh Narula, V. M. Kulkarni, Nalini Ramamurty, John Oommen, Ramesh S. Jadi, R. V. Banpel, Ana Maria Henao-Restrepo

**Affiliations:** 1 National Polio Surveillance Project, India Country Office, World Health Organization, New Delhi, India; 2 Vaccine Delivery, Bill & Melinda Gates Foundation, New Delhi, India; 3 Polio Operations & Research, World Health Organization, Geneva, Switzerland; 4 GAVI, The Vaccine Alliance, Geneva, Switzerland; 5 Department of Health and Family Welfare, Govt. of Maharashtra, Pune, Maharashtra, India; 6 Immunization & Vaccine Development, South-East Asia Regional Office, World Health Organization, New Delhi, India; 7 National institute of Virology, Indian Council of Medical Research, Pune, Maharashtra, India; 8 Immunization Vaccines and Biologicals, World Health Organization, Geneva, Switzerland; University of Massachusetts, United States of America

## Abstract

**Background:**

According to WHO estimates, 35% of global measles deaths in 2011 occurred in India. In 2013, India committed to a goal of measles elimination by 2020. Laboratory supported case based measles surveillance is an essential component of measles elimination strategies. Results from a case-based measles surveillance system in Pune district (November 2009 through December 2011) are reported here with wider implications for measles elimination efforts in India.

**Methods:**

Standard protocols were followed for case identification, investigation and classification. Suspected measles cases were confirmed through serology (IgM) or epidemiological linkage or clinical presentation. Data regarding age, sex, vaccination status were collected and annualized incidence rates for measles and rubella cases calculated.

**Results:**

Of the 1011 suspected measles cases reported to the surveillance system, 76% were confirmed measles, 6% were confirmed rubella, and 17% were non-measles, non-rubella cases. Of the confirmed measles cases, 95% were less than 15 years of age. Annual measles incidence rate was more than 250 per million persons and nearly half were associated with outbreaks. Thirty-nine per cent of the confirmed measles cases were vaccinated with one dose of measles vaccine (MCV1).

**Conclusion:**

Surveillance demonstrated high measles incidence and frequent outbreaks in Pune where MCV1 coverage in infants was above 90%. Results indicate that even high coverage with a single dose of measles vaccine was insufficient to provide population protection and prevent measles outbreaks. An effective measles and rubella surveillance system provides essential information to plan, implement and evaluate measles immunization strategies and monitor progress towards measles elimination.

## Background

Recent estimates indicate that global measles mortality has declined by 71% between 2000 and 2011 [1]. During the same period measles deaths in India declined by a mere 36% and still contribute to 35% of the global burden of measles deaths. India reported a little above 29 000 measles cases in 2011, which was certainly an underestimate. More recently, in September 2013, eleven countries of WHO South-East Asia (SEA) region, which includes India, have committed themselves to the goal of measles elimination by 2020 [2].

Given India's large burden of estimated measles cases and deaths, successful measles control efforts in the country are paramount to attaining regional and global measles elimination goals. India's existing strategy for measles control has the objective of mortality reduction and not elimination and as such it depends on measles outbreak surveillance rather than case based surveillance [3]. As India develops strategies for measles elimination, a case based measles surveillance system will likely form an integral part of its operational plans in order to inform decisions regarding appropriate strategy options, guide immunization activities and evaluate progress towards elimination.

In December 2009, the Measles Aerosol Vaccine Project (MAVP) of World Health Organization began a phase-II/III trial for measles vaccine administered as an aerosol in Pune district located in Maharashtra state [Clinical Trials Registry, India no.: CTRI/2009/091/000673 available at http://ctri.nic.in/Clinicaltrials/login.php]. Study subjects were recruited from 94 villages spread over three contiguous *Talukas* or Blocks (1^st^ sub-district level administrative division in India) — Haveli, Khed and Shirur of Pune. To systematically investigate and quantify the intensity of measles virus transmission in these three blocks of Pune included in the trial, it was deemed necessary to set up a case based surveillance system that would meet international performance standards and would run concurrently but independently of the trial. The National Polio Surveillance Project of WHO-India Country Office (WHO-NPSP) provided technical assistance to Government of Maharashtra to establish a case-based measles surveillance system in the MAVP Blocks (Unpublished document: WHO-NPSP Project Proposal for case based measles surveillance in MAVP Blocks of Pune). Concurrence from Government of India and Maharashtra state government was obtained to design and establish such a surveillance system. Apart from the immediate needs of the aerosol vaccine project in 2009, we believed that setting up this case based measles surveillance system in Pune would also serve as a model for scaling up later, as India takes on more aggressive measles control goals in future.

India had introduced one dose of measles vaccine between 9 and 12 months of age in its infant immunization programme in 1985. The latest evaluated coverage estimate for first dose of measles containing vaccine (MCV1) among infants in India was 74% [4]. Evaluated MCV1 coverage in Maharashtra state in India was 85% in 2007–08 and 91% in 2009. For Pune district, MCV1 coverage was 94% in 2007–2008 [5].

From 2010, India introduced a second dose of measles-containing vaccine (MCV2) through catch-up campaigns targeting children 9 months to 10 years of age in 14 states (which had MCV1 coverage below 80%) and through routine immunization programme for 16–24 month old children in 21 remaining states (with MCV1 coverage at or above 80%) including Maharashtra [6]. However, Maharashtra state had not introduced MCV2 in routine immunization until July 2011. In absence of reliable laboratory supported surveillance data it would be difficult to assess the impact of the catch-up campaigns (14 states) and the need for additional strategic interventions in the 21 states without such campaigns.

This paper describes the epidemiology of measles in three MAVP blocks of Pune for the period November 2009 through December 2011. Surveillance system performance is assessed against WHO internationally accepted indicators. Incidence rates and burden of measles and policy implications for measles control and elimination strategies for India are discussed. Design issues that were critical for the success of this case based surveillance system have been elaborated. We also discuss below why this model of case based measles surveillance can be taken as an example of legacy planning envisaged in the polio endgame strategic plan [7].

## Methods

### Population and area under measles surveillance

**Figure 1:**
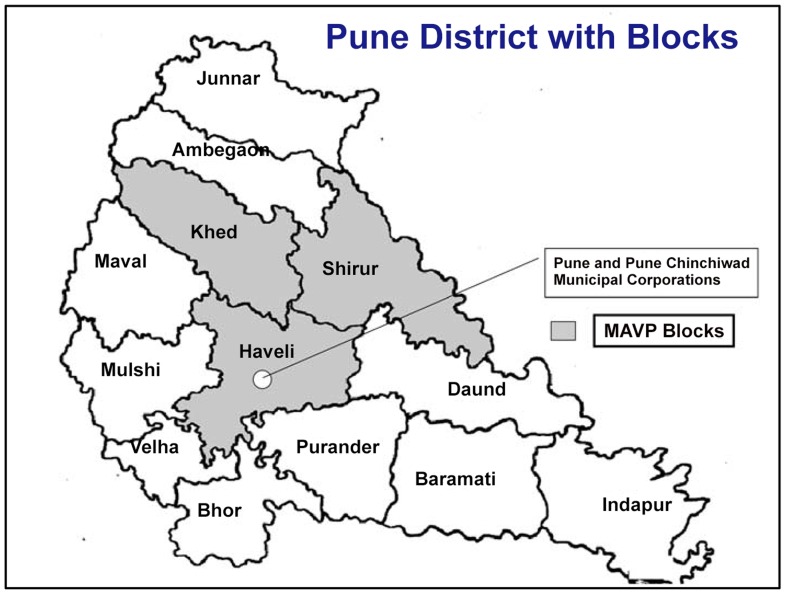
Pune district showing blocks under case based measles surveillance.

Figure 1 shows the area under surveillance in the three contiguous blocks, Haveli, Khed and Shirur of Pune district with a population of 1.37 million. These blocks comprised of 467 villages including the 94 villages from which subjects for the measles aerosol vaccine trial were recruited.

### Establishment of case based measles surveillance

Measles case-based surveillance was built on the platform of pre-existing network of reporting sites for Acute Flaccid Paralysis (AFP) surveillance for polio in Pune. In 2010, Pune district had 451 AFP reporting sites in the polio surveillance network. The vast majority of these reporting sites (85%) were private health care facilities. All the AFP reporting sites and some additional clinical care-givers were included in the network for measles surveillance and were required to submit AFP plus suspected measles case reports following a standard reporting protocol. Government and WHO staff conducted periodic surveillance workshops to sensitize and train clinical and other categories of personnel at the network reporting sites.

### Case definitions and case classification [8], [9]

A suspected measles case was defined as any person in whom a clinician suspected measles infection or any person with fever and maculo-papular rash with cough or coryza (running nose) or conjunctivitis (red eyes).

Based on laboratory and/or epidemiological criteria as described below, a suspected measles case was classified into one of the following categories

- **Laboratory confirmed measles or rubella case** if the person's serum sample tested positive for either measles or rubella IgM antibody;
- **Epidemiologically confirmed measles or rubella case** if the person's serum could not be tested in a laboratory for measles or rubella IgM (or returned equivocal results) but the case was related geographically and temporally (dates of rash onset within 21 days of each other) to another confirmed measles or rubella case (either laboratory-confirmed or epidemiologically confirmed);
- **Clinically confirmed measles case** if the person's serum was could not be tested in a laboratory (or returned equivocal results) and the case was also not epidemiologically linked to any other confirmed measles or rubella case;
- **Discarded case** if the serum sample tested negative for both measles and rubella IgM or if no serum sample was tested but the case was epidemiologically linked to an outbreak that was negative for both measles and rubella.

For outbreak classification, please see below.

In this report, laboratory, epidemiologically or clinically confirmed cases of measles have been collectively referred to as ‘confirmed measles cases’ and laboratory or epidemiologically confirmed rubella cases of rubella as ‘confirmed rubella cases’.

A measles death was defined as a death which occurred within 30 days of onset of rash in a confirmed measles case [10].

### Case reporting and investigation

For every suspected case of measles of any age resident in an MAVP block, a case investigation form (CIF) was completed and a blood sample was collected for serologic confirmation (either at the reporting site or at the home of the case).

All suspected cases were offered appropriate clinical care including therapeutic doses of vitamin A as per Govt. of India guidelines [11].

Each suspected measles case reported from a reporting site and resident in an MAVP block served to trigger a community search for additional suspected measles cases by public health staff. Additional cases detected were also investigated as described above or as described below if an outbreak was identified.

All suspected measles cases from MAVP blocks were followed up to ascertain their vital status (alive or dead) up to 30 days after onset of rash. Health workers visited home of case (or clinic if admitted) between 30 and 40 days after rash onset or earlier if death was reported before 30 days.

### Outbreak investigation

An outbreak was defined as a cluster of two or more suspected measles cases in a village in a week or if there was a continuous occurrence of cases every week over a 3–4 week period. Identification of a suspected outbreak prompted active searches in the community to identify additional cases by visiting all houses, supplementary nutrition centres for children, and schools in the village to detect more suspected measles cases of any age. Regular visits by District Health staff and WHO staff continued until there was a continuous period of three weeks during which no further cases were reported or the lab results showed that the outbreak was not due to measles [12].

Table **1** shows the protocol followed to classify outbreaks. When an outbreak was classified as measles or rubella outbreak based on laboratory results as per this protocol, the cases from that outbreak from whom blood samples had not been collected (see below for blood sample collection protocol) were classified as epidemiologically confirmed measles or rubella cases. If the outbreak was classified as a “mixed outbreak”, such cases were considered epi-linked to a mixed outbreak without ascribing the aetiology of the rash to either measles or rubella.

**Table 1:** Outbreak classification protocol.

Laboratory results of samples collected from an outbreak	Classification of Outbreak	Classification of cases in outbreak
≥2 measles positive and <2 rubella positive	Measles	Epidemiologically confirmed measles
≥2 rubella positive and <2 measles positive	Rubella	Epidemiologically confirmed rubella
≥2 measles positive and≥2 rubella positive	Mixed	Epidemiologically linked to mixed outbreak
<2 measles positive or <2 rubella positive	Discarded (negative measles and rubella)	Discarded

### Laboratory sample collection and case confirmation

Blood samples were collected through venepuncture from every suspected measles case that was reported as a sporadic case in any of the MAVP blocks. The system aimed at collecting one serum sample from every sporadic case within 28 days from onset of rash.

In the event of a suspected measles outbreak, blood samples were collected from the initial cases of an outbreak until at least 5 samples had been collected or at least 2 samples from that outbreak tested positive for either measles or rubella. The National Institute of Virology (NIV), Pune tested blood samples for measles immunoglobulin-M (IgM) through EIA with Enzygnost Anti-Measles-Virus/IgM as per WHO protocol. Samples testing negative for measles were tested for rubella IgM [13]. Measles laboratory of NIV Pune was independently accredited by WHO.

### Data compilation and sharing

Epidemiological data from surveillance and lab results from NIV laboratory in Pune were linked through a unique identifier assigned by the District Immunization Officer (DIO), Pune and WHO Pune unit of the WHO National Polio Surveillance Project (WHO-NPSP). WHO-NPSP central unit circulated summary tables and charts to relevant stakeholders monthly.

If an outbreak or a suspected measles case was reported from any of the villages from where subjects were recruited for the measles aerosol vaccine trial, DIO, Pune and/or WHO Pune unit immediately informed the local coordinator of the MAVP trial area.

### Statistical methods

Incidence rates for suspected measles, confirmed measles, confirmed rubella and discarded cases were calculated per 100 000 person-years. Case fatality ratio (CFR %) for confirmed measles cases was calculated as the ratio of measles deaths to the number of confirmed measles cases, expressed as a percentage.

Performance indicators for measles surveillance were calculated as recommended by WHO [8], [14]. Timeliness and completeness of reports of suspected measles cases from all the reporting units of Pune district were calculated on a weekly basis and were the same as reported to the AFP surveillance system.

The Kruskal-Wallis H test (One Way Analysis of Variance) was applied to compare the difference between medians and the Cornfield 95% confidence limits for odds ratio. Epi Info for Windows version 3.5.3 released January 26, 2011 was used for analysis.

### Ethics statement

The case based measles surveillance system in Pune was set up as part of public health disease surveillance of Govt. of Maharashtra and as such explicit review by an ethics committee was deemed unnecessary. Public health surveillance being a state issue, permission was sought and obtained from Additional Director of Health Services Govt. of Maharashtra before initiating surveillance. Govt. of India guidelines for measles surveillance were followed or adapted for all surveillance activities including blood sample collection and clinical care of suspected measles cases [11]. During outbreak investigations, verbal permission was obtained from suspected measles cases and/or their caretakers before collecting blood samples as per standard public health surveillance practice. If the case was admitted to a clinic or hospital, the surveillance team also obtained permission from the attending clinician to draw a blood sample.

## Results

The descriptive epidemiology of the measles cases identified from surveillance week 45 of 2009 (starting 1 November 2009) until week 52 of 2011 (ending 1 January 2012) is presented below (Table 2). In addition, performance of the surveillance system has also been assessed against global standards.

**Table 2:** Suspected measles cases by classification and incidence rates per 100 000 persons.

	2009^a^		2010		2011	
	Number	Incidence Rate^b^	Number	Incidence Rate	Number^c^	Incidence Rate
Suspected measles	21	9.21	474	34.53	516	36.76
Confirmed measles (Laboratory, epidemiologically or clinically confirmed)	17	7.46	376	27.39	379	27.00
Confirmed rubella (Laboratory or Epidemiologically confirmed)	1	0.44	18	1.31	39	2.78
Discarded	3	1.32	80	5.83	86	6.13

a : 2009: From week 45; 2010 and 2011: entire year.

b : Annualized Incidence rate per 100,000 persons per year;

c : 12 cases were epi-linked to a mixed outbreak of measles and rubella.

### Summary case counts by classification

In total, 1011 suspected measles cases were reported through the surveillance system. Of these, 169 (17%) were laboratory negative for both measles and rubella and discarded and 772 (76%) were classified as confirmed measles cases. Of confirmed measles cases, 509 were serum IgM positive, 228 were epidemiologically-linked in time and space to a lab confirmed or an epidemiologically confirmed measles case and 35 were confirmed by meeting the clinical case definition only. An additional 58 cases (6%) were confirmed rubella. Twelve cases (1%) were epidemiologically linked to an outbreak of both measles and rubella.

### Measles outbreaks

Frequent measles outbreaks characterised measles transmission in Pune. The surveillance system detected 21 suspected measles outbreaks of which 20 were subsequently laboratory confirmed as measles outbreaks (Table 3). One outbreak in 2011 was classified as a mixed outbreak of both measles and rubella cases (29 cases). Overall, 47% (362/772) of the confirmed measles cases occurred as part of measles outbreak and the rest were sporadic measles cases that did not occur in an outbreak setting.

**Table 3:** Suspected measles outbreaks with classification.

	2009^a^	2010	2011	Median no. of cases (IQR^b^)	Median Duration in days (IQR)
	No. of Outbreaks (cases)	No. of Outbreaks (cases)	No. of Outbreaks (cases)		
Suspected measles outbreaks	1 (7)	15 (267)	5 (117)	18 (8–26)	40 (22–68)
Confirmed measles outbreaks	1 (7)	15 (267)	4 (88)	17 (8–23)	39 (22–64)

a : 2009: From week 45; 2010 & 2011: entire year.

b : IQR: Inter-quartile range.

### Measles incidence and seasonal variation

**Figure 2:**
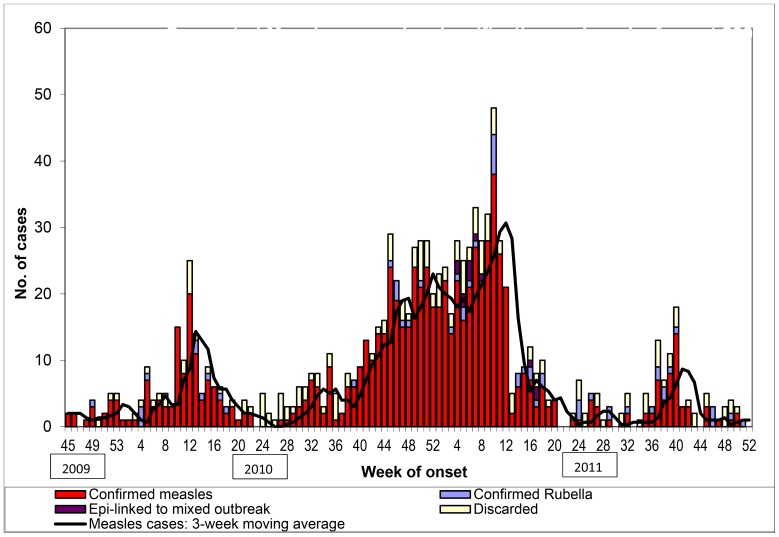
Suspected measles cases by week of onset and final classification: 2009–2011.

During the 113-week period under observation, confirmed measles cases occurred in 81% (91/113) of the weeks. Figure 2 shows persistent and regular cycles of measles transmission. Mean weekly incidence of confirmed measles cases was seven. However, measles incidence showed pronounced seasonal variation with the mean weekly incidence increasing to 20 cases for a 17-week period (week 44 of 2010 - week 8 of 2011) from November 2010 through February 2011. In Pune, these are the winter months when the average minimum monthly temperature remains at or below 15°Celsius [15]. For the two full calendar year periods (2010 and 2011) under surveillance, annual incidence rate of confirmed measles cases was greater than 250 per million persons.

### Age and sex distribution of cases by classification

Forty-five per cent of (349/772) of confirmed measles and 52% (30/58) of confirmed rubella cases were female. Table 4 shows the age distribution of confirmed measles, rubella and discarded cases. Of the 772 confirmed measles cases, only 4% occurred in children younger than 9 months of age (the recommended age of vaccination) and about 10% of cases occurred in infants. Most cases occurred in children 1–9 years of age (68%) while another 16% occurred before reaching 15 years of age and only 6% occurred in persons older than 15 years of age. In contrast, there were no confirmed rubella cases in infants. Of the 58 confirmed rubella cases, 72% were between 1 and 10 years and another 22% occurred before reaching 15 years of age. However, 6% of confirmed rubella cases occurred in persons older than 15 years demonstrating susceptibility to rubella during reproductive years. Compared to rubella, measles virus infected earlier in life (<10 year old) and even in infancy, while some residual susceptibility to both viruses remained beyond 15 years of age.

**Table 4:** Age distribution of confirmed measles, rubella and discarded cases, 2009–2011^a^.

	Confirmed measles cases (%)	Confirmed rubella cases (%)	Discarded cases (%)
<9 months	32 (04)	0 (00)	15 (09)
9–11 months	44 (06)	0 (00)	19 (11)
1–4 years	265 (34)	13 (22)	66 (39)
5–9 years	264 (34)	29 (50)	43 (25)
10–14 years	125 (16)	13 (22)	20 (12)
> = 15 years	42 (06)	3 (06)	6 (04)
Total	772 (100)	58 (100)	169 (100)

a : Confirmed measles: Laboratory, epidemiologically or clinically confirmed measles cases; Confirmed rubella: Laboratory or epidemiologically confirmed rubella cases; Discarded: Negative laboratory results for measles or rubella.

**Figure 3:**
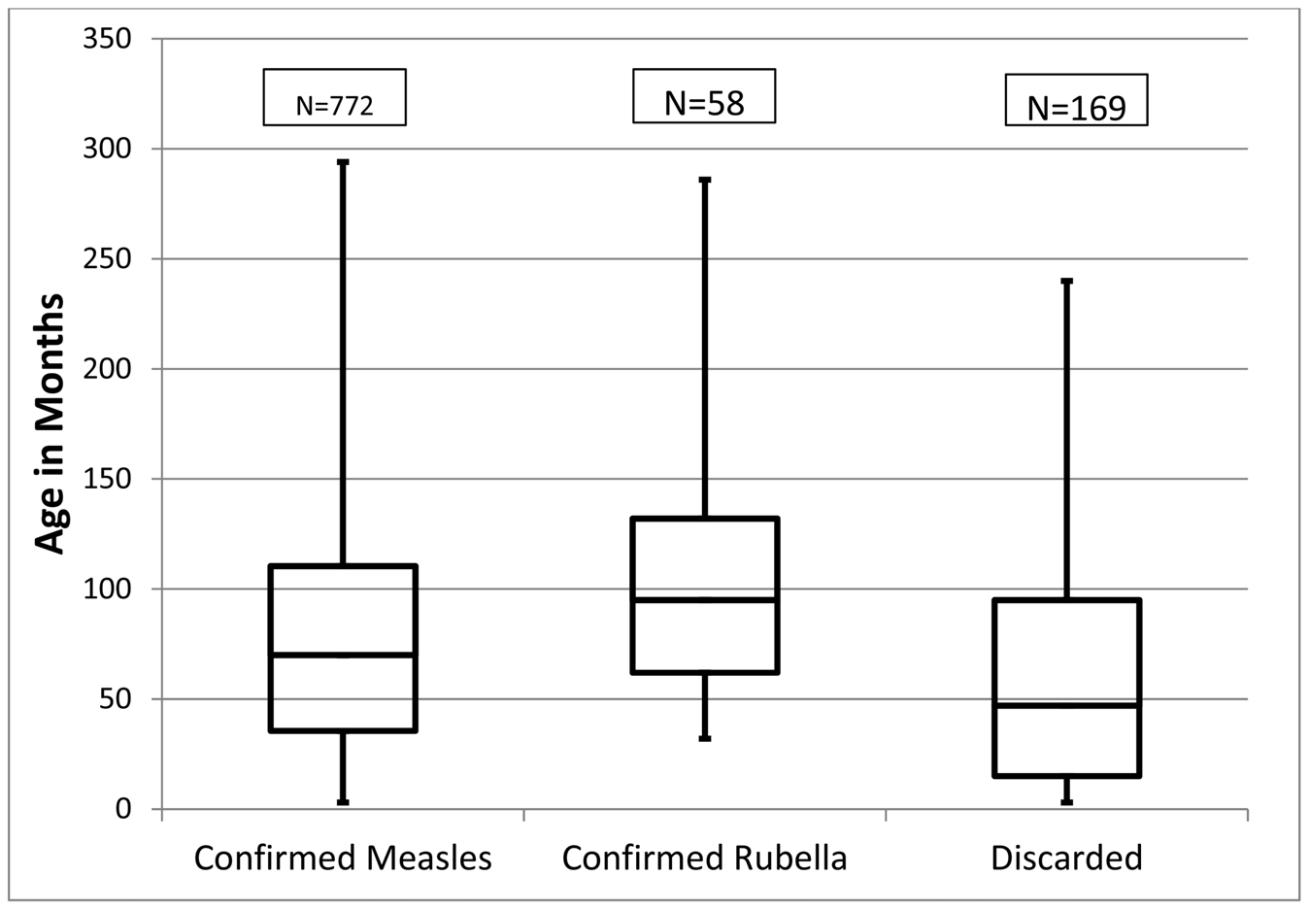
Confirmed measles, confirmed rubella and discarded cases: Box and whiskers plot for median, inter-quartile range, minimum and maximum ages (months).

The median age, inter-quartile range and the minimum and maximum age in months for confirmed measles cases (Median: 70; inter-quartile range: 36 to 111), for confirmed rubella cases (M: 95; IQR: 62 to 132) and for discarded cases (M: 47; IQR: 15 to 95) are shown in Figure 3. The differences between the median ages were statistically significant (Kruskal Wallis H = 28.429 at 2 degrees of freedom, p = 0.000001).

### Vaccination status of cases by classification

Overall 43% (437/1011) of the suspected measles cases reported vaccination with at least one dose of measles containing vaccine (MCV). Of the 437 cases who had a history of vaccination with MCV, only 98 (22%) had supporting documents (vaccination card, clinic prescription or vaccination register) in addition to historical recall, while the rest had no such supporting documents.

Of the 772 confirmed measles cases, 298 (39%) reported vaccination with at least one dose of MCV; of the 227 non-measles cases (confirmed rubella and discarded), 135 (59%) were vaccinated (Table 5). Only 34% of the confirmed measles cases aged 9–11 months had a history of measles vaccination compared to 68% of the discarded cases in the same age group. Similarly only 39% of the confirmed measles cases 1–4 years of age had history of measles vaccination compared to 67% of the discarded cases in the same age group.

**Table 5:** Number and proportion vaccinated a with measles containing vaccine by age-group and case classification b 2009–2011.

	Confirmed measles cases		Confirmed rubella cases		Discarded cases	
	Number	Proportion vaccinated	Number	Proportion vaccinated	Number	Proportion vaccinated
<9 months	32	6%	0	-	15	0%
9–11 months	44	34%	0	-	19	68%
1–4 years	265	39%	13	100%	66	67%
5–9 years	264	44%	29	76%	43	63%
10–14 years	125	41%	13	46%	20	40%
> = 15 years	42	26%	3	33%	6	17%
Total	772	39%	58	72%	169	55%

a : Vaccinated with at least 1 dose of measles containing vaccine.

b : Confirmed measles: Laboratory, epidemiologically or clinically confirmed; Confirmed rubella: Laboratory or epidemiologically confirmed; Discarded: Negative laboratory results for measles or rubella.

The odds ratio for vaccination with an MCV between a confirmed measles case and a non-measles case was 0.43 (Cornfield 95% confidence limits for odds ratio: 0.31 to 0.59).

### Case Fatality Ratio (CFR)

Of the 1011 suspected measles cases, 986 (98%) were followed up 30 days from onset of rash to ascertain vital status. Two of the 1101 cases died within 30 days of rash onset. Both were confirmed measles cases. The observed CFR in confirmed measles cases was 0.26% (2/772) and the 95% confidence interval for CFR was 0.00%–0.62%.

### Surveillance performance indicators

The measles surveillance system in Pune met globally recommended performance indicators for case based measles surveillance [8], [9].

Every week more than 80% of 451 reporting units submitted complete and timely reports (Table 6). Except for the first few weeks after start-up in 2009, the surveillance system consistently achieved a reporting rate above 2 per 100 000 persons for discarded cases and 70% of the suspected measles cases were investigated within 48 hours of notification. Over 80% of the suspected measles cases not linked to an outbreak had an adequate blood sample taken.

**Table 6:** Performance indicators for case based measles surveillance Pune, 2009–2011.

	2009	2010	2011
Number and completeness (%) of weekly reporting^a^	(4850/5486) 88%	(4559/5517) 83%	(4895/5587) 88%
Number and timeliness (%) of weekly reporting^a^	(4850/5486) 88%	(4559/5517) 83%	(4895/5587) 88%
Incidence of discarded cases per 100,000 persons in 3 MAVP blocks^b^^,^ c	1.3	5.8	6.1
Number and proportion (%) of suspected measles cases with an adequate sample^d^	(14/17) 82%	(278/295) 94%	(443/459) 97%
Number and proportion (%) of suspected measles cases investigated within 48 hours of notification^e^	(21/21) 100%	(390/474) 82%	(362/516) 70%

a : For the entire district of Pune;

b : For year 2009, indicator calculated as an annualized rate from week 45;

c : Target≥2 per 100,000 persons;

d : Target≥80% [Cases which were epidemiologically linked to an outbreak of measles, rubella or another infectious disease or to an outbreak of unknown aetiology excluded from denominator];

e : Target≥80%.

## Discussion

To our knowledge this is the first report from an active case based laboratory supported measles surveillance system in India. Earlier reports in India were mostly from special studies, outbreak investigations or from passive reporting [16], [17].

The World Health Assembly (WHA) in 2010 declared interim milestones towards measles elimination to be achieved by 2015 [18]. One of the three milestones is to reduce annual measles incidence to 5 per million persons in presence of adequate surveillance. Our data documents endemic measles transmission in Pune despite achieving evaluated coverage greater than 90% with one dose of MCV. For both 2010 and 2011, the annual measles incidence observed in Pune was more than 50 times (∼270 per million persons) above the interim targets set by WHA.

Reported measles incidence rate for India in 2011 was 24 per million persons [1]. Given that the national MCV1 coverage for India was 74% in 2009, lower than that evaluated for Pune (94%), this likely underestimates the true incidence of measles in India [4], [5].

Simons et al. note the lack of reliable case based measles surveillance data as a constraint in deriving realistic modelled estimates of incident measles cases and deaths in India. Their model estimated 65 500 (95% CI: 53 600–78 800) measles deaths in India in 2010 [19]. Another retrospective sample survey of cause-specific child mortality through verbal autopsies estimated 92 000 (99% CI: 79 000–104 000) measles deaths in India in 2005 [20]. A recent review of community based studies in India determined median case fatality ratio (CFR) for measles at 1.63% (IQR: 0%–5%) [21]. However, reported measles cases and deaths are usually much lower, 48 181 cases and 188 deaths in 2008 [22].

Owing to paucity of reliable laboratory confirmed surveillance data, estimates of measles cases and deaths for India are thus affected by large levels of uncertainty. By providing additional population based estimates of confirmed measles incidence, our data will possibly contribute to more robust and accurate estimates of measles diseases burden in India.

In September 2013 India became a party to the WHO South East Asia Regional declaration for measles elimination by 2020 [2]. However, India's measles control programme is still focused on measles mortality reduction rather than measles elimination [3]. As India starts implementing its measles elimination programme, a sensitive case based surveillance system will be essential to guide immunization activities and monitor progress towards the goal of elimination.

As of 2013, 11 states of India maintain laboratory supported measles outbreak surveillance system with technical assistance from WHO-NPSP. However, there is no national system for laboratory supported case based measles surveillance. As India has just completed the first round of measles catch-up campaigns in 2013, targeting 134 million children in 14 of its 35 states, transitioning to laboratory supported case based measles surveillance system will be necessary to substantiate the impact of the intervention in these states [23]. To guide future measles control and/or elimination strategies it would also be necessary to set up case based measles surveillance systems in all other states of India.

At the time of this report, Pune district had not introduced a second dose of MCV in its immunization program. Despite high coverage (>90%) achieved with a single dose of measles vaccine through routine immunization, endemic measles transmission with periodic measles outbreaks continued to occur with nearly half of confirmed measles cases associated with outbreaks. These findings demonstrate that high level of vaccination coverage with one dose of measles vaccine is insufficient to reach threshold levels of herd protection required to interrupt measles transmission. This locally derived empirical evidence provides additional support to current Government of India policy and WHO guidance for the need to sustain high coverage with two doses of MCV for sustained measles control [24]. Experience from several countries provide corroborative evidence that high coverage with two doses of measles vaccine is needed to sustain measles control and interrupt transmission [25].

We also explored whether lowered effectiveness of one dose of measles vaccine under field conditions could be the cause of continued measles transmission and outbreaks in Pune. In our data, 39% of the confirmed measles cases in the 1–4 year age group were vaccinated. Applying the Orenstein curves and equations to our observed data of 39% of cases vaccinated (PCV) and 94% of population vaccinated (PPV) with MCV1, estimated measles vaccine effectiveness is 96% [26]. This is well within the range of estimated measles vaccine efficacy of 92% (IQR: 84%–96%) at 9 months and 99% (IQR: 93–100%) at 11 months of age [27].

In Pune district, 78% of measles disease burden is borne by children under 10 years and 95% of cases occur by 15 years of age. Data from measles outbreak surveillance in other states of India with MCV1 coverage equal or greater than 85% show a similar pattern. In Tamil Nadu and Kerala, 80% and 71% of cases respectively, occurred in children below 10 years of age in 2010 [28].

This has important policy implications. Between 2010 and 2013, India has implemented large scale measles catch-up campaigns to vaccinate children between 9 months and 10 years of age in 14 (out of 35) states with MCV1 coverage less than 80% [23]. For the 21 remaining states (including Maharashtra) with relatively higher MCV1 coverage, which have not undertaken catch-up campaigns so far, the immunization programme will also need to take into account measles disease burden and susceptibility in older age cohorts (beyond 10 years) to reach the WHA recommended interim goal of measles incidence of five per million persons.

Several limitations to our data should be noted. The reporting network was geographically restricted to the district of Pune. Measles cases (from the three MAVP blocks) that sought clinical care at sites outside Pune district might well have been missed. In India, as in other countries, the number of measles cases and associated deaths may be underreported as measles cases may not seek treatment at medical care facilities or cases are not reported through the surveillance systems [29], [30]. Except when doing an outbreak investigation, we did not routinely canvass households for suspected measles cases and might thus have missed some cases. On the other hand, including clinical care providers within Pune, from both public and private sectors (85% of reporting sites) in our surveillance network ensured that cases attending either type of health facility in the district were reported to the surveillance system.

Case-based measles surveillance achieved most of the globally recommended cardinal indicators of measles surveillance performance (Table 6) including Incidence of discarded cases higher than 2 per 100,000 persons and more than 80% suspected measles cases with an adequate serum sample [8]. While it is believed that the effectiveness and sensitivity of measles case-based surveillance in Pune benefited from its link with highly sensitive polio AFP surveillance, it cannot be assumed that all cases were detected. Unfortunately, we did not test the sensitivity of the surveillance system through a capture-recapture of cases or other methods and are therefore unable to compare the epidemiological characteristics (e.g. age, vaccination status etc.) of suspected cases missed by the reporting system vs. those that were reported. The surveillance system made follow-up visits to ascertain vital status up to 30 days after onset of rash but did not systematically record all complications occurring between onset and the follow-up visit.

In summary, the Pune case based measles surveillance system was built on the pre-existing sensitive and robust system of AFP surveillance for polio. Close coordination between Govt. staff and Surveillance Officers of WHO-NPSP, periodic sensitization workshops conducted for clinicians and public health staff, and active search for suspected measles cases in health facilities and in the community during outbreaks were critical elements in the success of the surveillance system. Operationally, this was similar to the surveillance system in place since the early 1990's in the Region of the Americas for measles surveillance in an elimination setting [31], [32].

This surveillance system was a tangible example of how polio resources can be leveraged to support other vaccine preventable disease control activities. WHO-NPSP has a country-wide network of Surveillance Medical Officers supporting Government of India and state governments in polio and measles outbreak surveillance. The experience of establishing and supporting this surveillance system in Pune with the direct involvement and collaboration between Government staff and the WHO-NPSP polio surveillance network can be replicated relatively easily in other states of India to establish measles case based surveillance to monitor progress towards measles elimination and inform immunization policy.

Globally, opportunities should also be explored to transition the existing polio eradication infrastructure and trained human resources to support broader immunization strengthening activities including integrated surveillance systems for measles and other vaccine preventable diseases. This is a concrete example demonstrating the feasibility of legacy planning objective of the 2013–2018 Polio Endgame Strategic Plan [7].
